# Elevated Temperature May Affect Nectar Microbes, Nectar Sugars, and Bumble Bee Foraging Preference

**DOI:** 10.1007/s00248-021-01881-x

**Published:** 2021-10-01

**Authors:** Kaleigh A. Russell, Quinn S. McFrederick

**Affiliations:** Department of Entomology, University of California, Riverside, Riverside, CA 92521 USA

**Keywords:** Floral microbes, Pollination, Symbiosis, Fructophilic bacteria

## Abstract

Floral nectar, an important resource for pollinators, is inhabited by microbes such as yeasts and bacteria, which have been shown to influence pollinator preference. Dynamic and complex plant-pollinator-microbe interactions are likely to be affected by a rapidly changing climate, as each player has their own optimal growth temperatures and phenological responses to environmental triggers, such as temperature. To understand how warming due to climate change is influencing nectar microbial communities, we incubated a natural nectar microbial community at different temperatures and assessed the subsequent nectar chemistry and preference of the common eastern bumble bee, *Bombus impatiens*. The microbial community in floral nectar is often species-poor, and the cultured *Brassica rapa* nectar community was dominated by the bacterium *Fructobacillus*. Temperature increased the abundance of bacteria in the warmer treatment. Bumble bees preferred nectar inoculated with microbes, but only at the lower, ambient temperature. Warming therefore induced an increase in bacterial abundance which altered nectar sugars and led to significant differences in pollinator preference.

**Supplementary Information:**

The online version contains supplementary material available at 10.1007/s00248-021-01881-x.

## Introduction

Rapid increases in global temperature have the potential to disrupt many ecological processes. Warming due to climate change has caused an increase of 0.85 °C in global surface temperatures over the past century [1]. Models based on low CO_2_ emissions estimate that there will be another 1.5 °C rise by the turn of the century [2]. Sixteen of the 17 hottest years in the past 138 years have occurred in the 2000s with 2016 and 2020 being the hottest years on record [1]. Extreme climatic events, including drought and heat waves, are also predicted to become more common [3].

Most living organisms, including microorganisms, have an optimal living temperature at which they thrive [4, 5]. As global temperatures increase, many species interactions will be disrupted [6]. Although there is ample research on plant phenological and physiological change in the context of climate change, there currently is a lack of information on how climate change will influence the interaction between flowers, pollinators, and their associated microorganisms. The center of this interaction web is floral nectar. Pollinators rely on nectar as a valuable source of energy [7]. However, nectar is more than a sugar-rich resource, and surveys of many wild plant species in varied ecological regions have revealed nectar is often inhabited by bacteria [8, 9] and fungi (mainly yeast) [10].

Nectar-inhabiting microorganisms have been found to alter nectar chemistry and influence pollinator behavior [11]. Specialized nectar-inhabiting microbes can tolerate the harsh nectary environment that filters out non-specialist microbes [12]. These specialized microbes utilize resources within floral nectaries and change nectar chemistry in many ways. For example, yeasts and bacteria change nectar sugar concentrations as they metabolize nutrients [13] as well as release volatile organic compounds which affect pollinator preference for nectar [14, 15]. Although fungi and bacteria can reduce overall nectar sugars when compared to sterile nectar, bees tend to prefer nectar colonized with specific microbes [13, 16]. However, microbe-mediated pollinator preference is dependent on pollinator species, the microbes present, and even gustatory and olfactory cues, which in some cases lead to pollinators showing indifference or even avoiding nectar colonized with less attractive microbes [14, 15, 17]. These microscopic changes in nectar lead to drastic changes in pollinator behavior, however, little is known about how climate change will affect this interaction.

With the looming prospect of rapid temperature increases, understanding the effects of climate change on plant-pollinator-microbial interactions is of great importance. We hypothesize that environmental temperature mediates microbial community structure in nectar because different microbes are likely to have different optimal growth temperatures. We predict that nectar-inhabiting microbial communities will differ between temperature treatments, leading to altered nectar chemistry and ultimately differences in pollinator preference. Here we test the effects of three different temperature treatments, representing baseline temperatures and climate change predicted temperatures, on the same starting microbial community using synthetic nectar. We determine how temperature-mediated changes in microbial communities alter nectar chemistry and pollinator preference. Our results help tease apart the mechanisms within plant-pollinator-microbe interactions that will be affected by climate change.

## Methods

### Brassica rapa and Nectar Extractions

*Brassica rapa* subsp. *oleifera*, Brassicaceae, is a wild mustard introduced to North America from Europe which is pollinated by many invertebrate species. We selected *B. rapa* as it is abundant in Southern California and is visited by many different pollinator species. We collected *B. rapa* flowers in the early morning (between 8:00 and 10:00am) from a meadow in Beaumont, California (33.933670, − 117.002738) in April 2017 and brought the flowers back to the lab for nectar extractions. Although the flowers were not bagged, we collected them early in the morning before many pollinators were out foraging. While we collected nectar early in the morning to control for pollinator visitation and microbial degradation, the nectar could have already been inoculated with sugar-altering microorganisms. The collection site was a mature field with all plants in the flowering stage. *Brassica rapa* inflorescences have 2–5 flowers open at a time, and we gently removed all unopened floral buds prior to nectar extraction. To avoid pollen and pollen microbes contaminating the nectar, we used sterile micro-dissecting scissors to carefully remove anthers and pollen from each flower. To extract nectar, we placed 2–5 flowers facing down in a sterile, modified 1.5 mL Eppendorf tube and centrifuged the flowers at 7500 × g for 1 min to remove nectar. We repeated this process with about 20 *B. rapa* inflorescences from five different plants until we had 25µL of nectar. To prevent any debris or small invertebrates from falling into the nectar during centrifugation, we modified the 1.5 mL Eppendorf tubes by gluing fine mesh halfway up the tube. We used centrifugation as the method to collect nectar as the *Brassica* flowers are too small to use capillary tubes. Although this method may introduce microbes from the petals or bracts it is unlikely that these microbes would thrive in the artificial nectar as they are phyllosphere bacteria [18]. We sterilized all 1.5 mL collection tubes with mesh modifications by UV (254 nm) sterilization in an AirScience UV-Box (Fort Meyers, FL) for 20 min prior to use. We pooled the nectar from the 20 *B. rapa* inflorescences from five different plants for chemical composition analysis and microbial community characterization.

### Nectar Analysis

To quantify nectar sugars, we used the Megazyme Sucrose, D-Fructose, D-Glucose Assay Kit and followed the manufacturer’s protocol. To identify the amino acid composition of the nectar samples, we sent *B. rapa* nectar to Texas A&M University Proteomics department where there is an established free amino acid assay for plant nectar using high-performance liquid chromatography (HPLC, Online Resource 1). Based on these two analyses of *B. rapa* nectar, we designed sterile, synthetic nectar by autoclaving a solution of 7.4% w/v molecular grade sucrose, 5.8% w/v molecular grade glucose, and 1.1% w/v molecular grade fructose for a roughly 7:5:1 ratio—which is similar to what was found in *B. rapa* nectar in Wykes (1952) [19]. We then added 8 mL of Minimum Essential Medium (MEM) sterile Non-Essential Amino Acid Solution (100 ×) (Sigma-Aldrich M7145), which contains seven of the highest registered amino acids from *B. rapa* nectar. We added amino acids to replicate the nectar as closely as possible to promote field-realistic microbial growth.

### Choice Experiment

We inoculated synthetic nectar with nectar-inhabiting microbial communities from wild *B. rapa* flowers by adding 50µL of pooled, freshly extracted *B. rapa* nectar from roughly 45 inflorescences (as described above) to 10 mL of synthetic nectar and allowed 24 h for growth at 25 °C. Then we gently vortexed this single pool of inoculated artificial nectar and added 10µL to 110 individual 2 mL tubes of synthetic nectar. We then separated these tubes into two treatments of 55 tubes each and incubated each treatment at one of two temperature treatments. The first temperature treatment (27 °C) represents the average spring-time high in Riverside, CA [20], where the nectar microbes were collected, and the second treatment (32 °C) represents a climate change predicted temperature [21]. High CO_2_ emission scenarios predict a 5 °C increase in global temperatures by the turn of the century [21]. We incubated treatments for 3 days, which is the longest that nectar will sit in the nectary of *Brassica* before being depleted or the flower senesces [22]. For controls, we also incubated 55 tubes of sterile synthetic nectar at each of the temperature treatments. After the 3-day incubation, we divided each volume of the mature microbial communities into two aliquots: one for characterization of the microbial community and nectar sugars, and one for the bumble bee choice assay.

We presented 1–5-day-old female worker *Bombus impatiens* with a choice assay to assess feeding preference. We purchased five *B. impatiens* colonies from Koppert Biological Systems (Howell, MI) and maintained these colonies with pollen and 60% sterile sucrose water ad libitum in environmentally controlled rooms at the University of California Riverside, which were held at 27 °C. We assayed 10 bumble bees from each colony for a total of 50 bumble bees. To perform the choice assay, we put one bumble bee in a small foraging chamber (9.5 cm × 10.15 cm) that had access to 4 feeders each filled with 1 mL of one of four treatments: (1) synthetic nectar inoculated with microbes and incubated at 27 °C, (2) sterile synthetic nectar incubated at 27 °C as a control, (3) synthetic nectar inoculated with microbes and incubated at 32 °C, and (4) sterile synthetic nectar incubated at 32 °C as a control. We point out that the temperature treatments occurred before the choice assay; all 4 nectar treatments were offered to bees at the same temperature as the bees. The bees themselves were not exposed to any temperature treatments but instead held at a constant 27 °C.

To assess bumble bee preference, we carefully weighed each feeder before and after a 24-h foraging period and counted choice as amount in grams of nectar consumed by each bee. We set up five control pots that had all four nectar treatments but no bumble bees. To account for evaporation, we weighed the feeders before and after the foraging period and subtracted the amount evaporated from the amount consumed by bees for each block. Each bee participated in only one choice test and was not returned to the colony after the assay was completed. To be sure that the experimental bees were not acclimated to any of the offered treatments, we did not train the bees to the assay arenas or to the artificial nectar, thus the experimental bees were naive to all four offered treatments in the choice assay. As experimental bees were reared in their respective colonies, it is possible that they were acclimated to hive microbial contaminants in the sugar water while in the colony. However, the lab colonies we used for these experiments were never exposed to environmental microbes and we regularly changed their sterilized sucrose solution. The shared sucrose source in the colonies would therefore only be exposed to the specialized bumble bee gut microbiota, which is closely related to the honey bee microbiota and is not known to grow in sucrose-only media [23]. However, previous work described the commonly found microbes in commercial bumble bee microcolonies [24] and none of these microbes were detected in our nectar treatment. Exposure of our experimental bees to nectar-inhabiting microbes before the choice trials is therefore highly unlikely if not impossible.

### Extreme Heat Experiment

We conducted a second choice assay using the same methods as described above but with more extreme temperatures. Using the *B. rapa* synthetic nectar recipe, we inoculated the same wild *Brassica* flower microbial community and incubated the nectar for 3 days. In this experiment, we used 32 °C and 42 °C to incubate the nectar before offering it to 50 bumble bees from 5 colonies (these colonies differed from those used in experiment above) in a choice assay as described above. We selected these temperature treatments as they represent the average summer daytime high in the geographical region and a typical heatwave temperature increase.

### Post-Assay Nectar and Microbiota Analysis

To characterize microbial communities, we centrifuged the aliquoted sample at 4500 × g for 5 min to pellet out microbes. Once the pellet was formed, we pipetted nectar off for carbohydrate analysis, leaving the pellet for DNA extraction. To measure carbohydrate concentrations on a subset of samples, 10 samples from each treatment, we again used the Sucrose, D-Fructose, D-Glucose Assay Kit (Megazyme, Chicago, IL) to characterize differences in nectar sugars according to temperature treatments.

We extracted DNA from the remaining pellet of all 100 samples using the DNeasy Blood and Tissue Kit (Qiagen, Valencia, CA). To control for possible reagent contaminants, we included *N* = 1 “blank” samples that contained no cells beyond those that may have occurred in the reagents or via possible contamination. We ran this blank sample through all of our library preparation and analysis pipeline. To prepare the samples for extraction, we used a Qiagen tissue lyser to bead-beat samples for 6 min at 30 Hz with two sterile 3.2 mm chrome-steel beads and roughly 100µL of 0.1 mm glass beads (Biospec, Bartlesville, OK), in 180µL of buffer ATL from the Qiagen extraction kit. We then added 20μL of Proteinase K, incubated the samples overnight at 57 °C, and followed the DNeasy standard extraction protocol.

To characterize the microbial communities within nectar, we used dual-index inline barcoding to prepare samples for sequencing on the MiSeq sequencer (Illumina), following the same protocols as detailed in McFrederick and Rehan (2016) [25]. We used primers that included either the forward or reverse Illumina sequencing primer, a unique 8-nt-long barcode, and the forward or reverse genomic oligonucleotide [26]. We used the bacterial 16S rRNA sequence primers 799F-mod3 CMGGATTAGATACCCKGG [27] and 1115R AGGGTTGCGCTCGTTG [26] and the fungal internal transcribed spacer (ITS) primers ITS1F (50-CTTGGTCATTTAGAGGAAGTAA-30) and ITS4R (50-TCCTCCGCTTATTGATATGC-30). We performed PCRs using 10μL of 2 × Pfusion High-Fidelity DNA polymerase (New England Biolabs, Ipswich, MA), 10μL of ultrapure water, 0.5μL of each 10 μM primer stock, and 4μL of DNA, with an annealing temperature of 57 °C for 30 cycles. We cleaned this product using Ultraclean PCR cleanup kit (MoBio, Carlsbad, CA), to remove unincorporated primers and dNTPs. To complete the Illumina sequencing construct, we used 1μL of the clean PCR product as a template for a second PCR, using HPLC-purified primers: CAAGCAGAAGACGGCATACGAGATCGGTCTCGGCATTCCTGC and AATGATACGGCGACCACCGAGATCTACACTCTTTCCCTACACGACG [26]. We then normalized 18μL of PCR product using SequalPrep Normalization plates (Thermo Fisher Scientific, Waltham, MA). We pooled 5μL of each sample and performed another Ultraclean PCR cleanup on this combined sample. We assessed library quality using a 2100 Bioanalyzer (Agilent, Santa Clara, CA). After quality control, we sequenced the libraries using a MiSeq sequencer (Illumina) and MiSeq Reagent kit, version 3 (Illumina), with 2 × 300 cycles, at the IIGB Genomics Core, UC Riverside. We were unable to amplify fungi from our artificial nectar samples, and therefore do not consider fungi further.

### Quantification of the Microbial Community (qPCR)

To determine the absolute abundance of bacterial cells in the artificial nectar samples of both temperature treatments, we used quantitative real-time PCR (qPCR) of the inoculated DNA extractions as above, *N* = 100; however, 30 samples failed to amplify (*N* = 70). Each PCR reaction consisted of 7.5μL SsoAdvanced master mix (Bio-Rad Laboratories, Los Angeles, CA), 3.6 mL molecular grade water, 0.45μL forward primer, 0.45μL reverse primer and 1.0 μl sample or standard DNA. We used the universal bacterial primers for the conserved 16S rRNA region Univ331F as our forward primer (5′-TCCTACGGGAGGCAGCAGT-3′) and Univ797R as our reverse primer (5′-GGACTACCAGGGTATCTAATCCTGTT-3′) [28]. We used this 16S qPCR primer set instead of 799F-1115R due to its established use in previous microbiome studies [28–31]. The reaction conditions were an initial heating at 95 °C for 3 min, followed by 39 cycles of 95 °C for 10 s, 59 °C for 30 s on a BioRad C1000 Touch thermal cycler. We compared our samples to a standard curve of 1 × 10^2^–1 × 10^8^ copies of the 16S rRNA gene cloned into a TOPO-TA plasmid (Invitrogen, Carlsbad, CA), with all qPCR efficiencies between 90 and 100% and *R*^2^ above 0.99.

### Statistical Analysis and Microbiome Bioinformatics

We used a two-way analysis of variance (ANOVA) and TukeyHSD post hoc pairwise comparison to assess whether temperature treatment affects individual nectar sugar concentrations and a Kruskal–Wallis chi-square test to test for differences in total sugars. We used a linear mixed model with Gaussian error distribution (GLMMs; package lme4) [32] to assess differences in nectar consumption by bumble bees during the choice assay. We used nectar consumption as the response variable, temperature treatment and presence or absence of a microbial community as fixed effects, and colony of origin as random intercept. We used package lmerTest to compare coefficients of fixed effects [33]. To test if 16S rRNA gene copy numbers (qPCR results) were different between temperature treatments, we used Welch’s *t*-test. We performed all the above statistical analyses in R 3.4.4 [34]. We used QIIME2-2018.6 [35] to process the 16S rRNA gene sequence libraries. We trimmed the low-quality ends off the reads with QIIME2’s default settings. Next, we binned our sequences into amplicon sequence variants (ASVs) using DADA2 [36], followed by chimera removal using the default settings of the DADA2 Pipeline. To assign taxonomy to the ASVs we used the QIIME2 q2-feature-classifer [37] trained to the 799–1115 region of the 16S rRNA gene and conducted local BLASTn searches against the NCBI 16S microbial database (July 2017). We cleaned the data by filtering out ASVs from the resulting feature table that corresponded to contaminants of reagents as identified in our blanks (Online Resource 2). As the artificial nectar in which the floral microbiomes were incubated contained no plant material, we found no plant plastid contamination in our sequencing reads. To generate a phylogenetic tree of our sequences, we used the MAFFT aligner [38] and FastTree v2.1.3 [39]. We used rarefaction analysis to determine a standardized coverage of bacterial species diversity to be used in alpha and beta diversity analyses of the DNA extracted from synthetic nectar after incubation period. At 2600 reads per sample we found that the rarefaction curves levelled off, and 63 samples could be included in the subsequent analyses. We used this tree and ASV table for alpha diversity analysis and to calculate unweighted UniFrac distance matrices in QIIME2 using qiime diversity core metrics. We used the Shannon Diversity Index and the Kruskal–Wallis test in QIIME2 to analyze alpha diversity. We analyzed differences in beta diversity with temperature treatment as a fixed factor using Adonis (type II sum of squares) with the vegan package in R [40].

## Results

### Sugar Changes with Temperature and Microbes

**Fig. 1:**
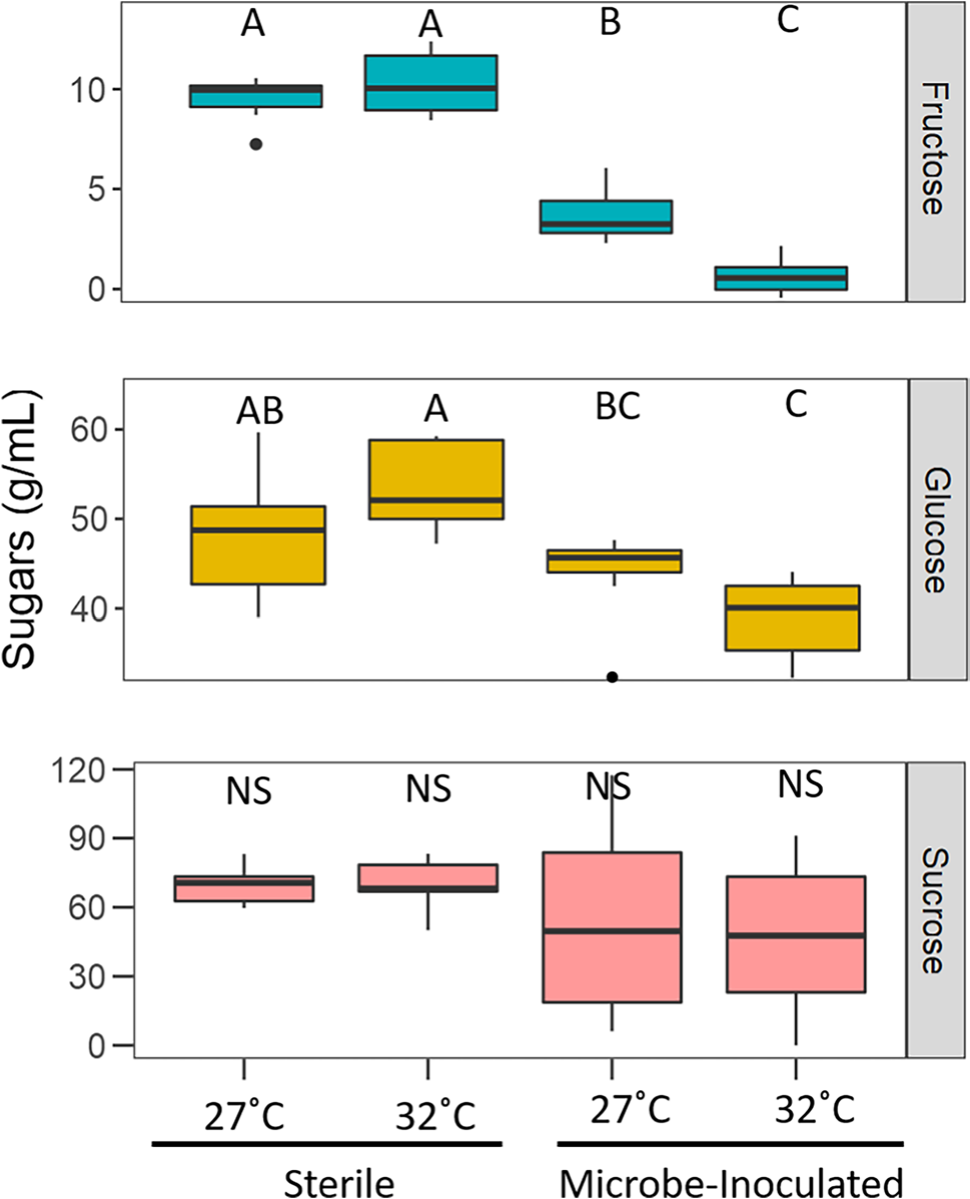
Boxplot indicating amounts of the three sugars after a 3-day incubation. There was more fructose in the in the 27 °C compared to 32 °C and significantly more in the sterile nectar compared to nectar with microbes. There were differences in glucose amounts between 32 °C sterile treatment, 27 °C microbe-inoculated treatment, and 32 °C microbe-inoculated treatment. There was no significant difference in amount of sucrose between treatments. Statistical difference is indicated by letters, NS, no significance

After the 3-day incubation period, artificial nectar sugars were significantly reduced by 9.5% when microbes were present (Kruskal–Wallis chi-square = 13.391; *N* = 50, *p* < 0.0001). As microbes consume nectar resources, overall nectar sugars decreased in the inoculated treatments compared to sterile nectar. Temperature treatment and presence of microbes influenced individual sugar concentrations. There was no significant difference in sucrose levels between any of the four treatments (Kruskal–Wallis chi-square = 2.7792, df = 3, *p* = 0.4269; Fig. 1). There was an interaction effect of temperature and treatment on fructose levels (F_1,36_ = 38.8, *p* < 0.0001; Fig. 1). There was roughly 50% more fructose in the in the 27 °C compared to 32 °C (Tukey HSD, *p* < 0.0001), and 75% more in the sterile nectar compared to nectar inoculated with microbes (Tukey HSD, *p* < 0.0001). There was also an interaction effect between temperature and treatment on glucose levels (F_1,36_ = 10.505, *p* = 0.002; Fig. 1) with significant differences in glucose amounts between 32 °C sterile treatment (Tukey HSD, *p* = 0.003), 27 °C microbe-inoculated treatment (Tukey HSD, *p* = 0.001), and 32 °C microbe-inoculated treatment (Tukey HSD, *p* < 0.0001).

### Bumble Bee Preference

**Fig. 2:**
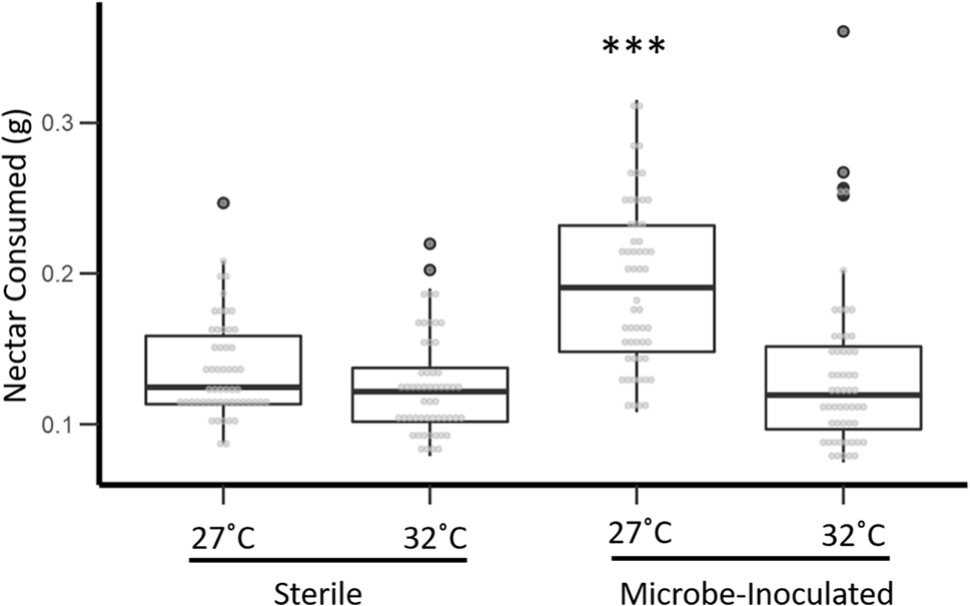
Mass of nectar consumed by bumble bees during the 24-h choice assay. Bumble bees were given the choice between four treatments. For the two incubation temperatures, there were a sterile control and a treatment (inoculated with a microbial community). Asterisk (***) indicates statistical significance

*Bombus impatiens* consumed significantly more nectar inoculated with microbes (GLMM; *t* = 6.854, *df* = 207, *p* < 0.0001, Fig. 2) and incubated at 27 °C (GLMM; *t* =  − 4.190, *df* = 207, *p* < 0.0001, Fig. 2), than all other nectar choices. Bumble bees preferred nectar inoculated with microbes and incubated at 27 °C 1.5 × more than synthetic nectar without microbes or nectar with microbes but incubated at 32 °C. We found no effect of evaporation on overall choice assay results. Similarly, in our “extreme heat” study, bumble bees preferred nectar incubated at lowest of the two temperature treatments (Online Resource 3). In both studies, bumble bees preferred nectar with a microbial community yet reared at the lower of the two temperatures.

### Microbial Community

**Fig. 3:**
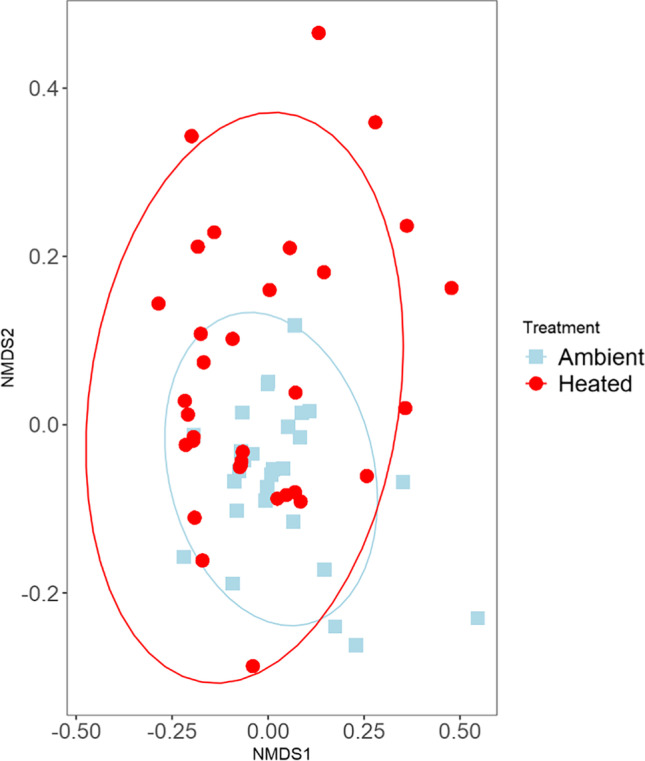
Nonmetric multidimensional scaling plot of the unweighted UniFrac distance matrices of synthetic nectar inoculated with a microbial community and incubated at two temperature treatments. Blue squares indicate 27 °C (ambient) treatments and red circles denote 32 °C (heated) treatments. Colored ellipses designate 95% confidence intervals around the centroid median of the points

There was a total of 397,885 quality-filtered reads with an average of 5604 reads per sample (*N* = 63) that clustered into 205 filtered ASVs for bacterial sequencing. We found that there was no significant difference in alpha diversity, using the Shannon Diversity Index, between temperature treatments (Shannon’s *H* = 1.3532, *p* = 0.244). Non-metric Multidimensional Scaling (NMDS) analysis on the unweighted UniFrac distance matrix (Fig. 3) showed that there was no obvious clustering by treatment. We analyzed the unweighted UniFrac distance matrix of our samples with the Adonis function in the R package vegan [40] (999 permutations PerMANOVA) using temperature as an explanatory variable and found no significant difference between temperature treatments (*F* = 1.0562, *R*^2^ = 0.02344, *p* = 0.32).

**Fig. 4:**
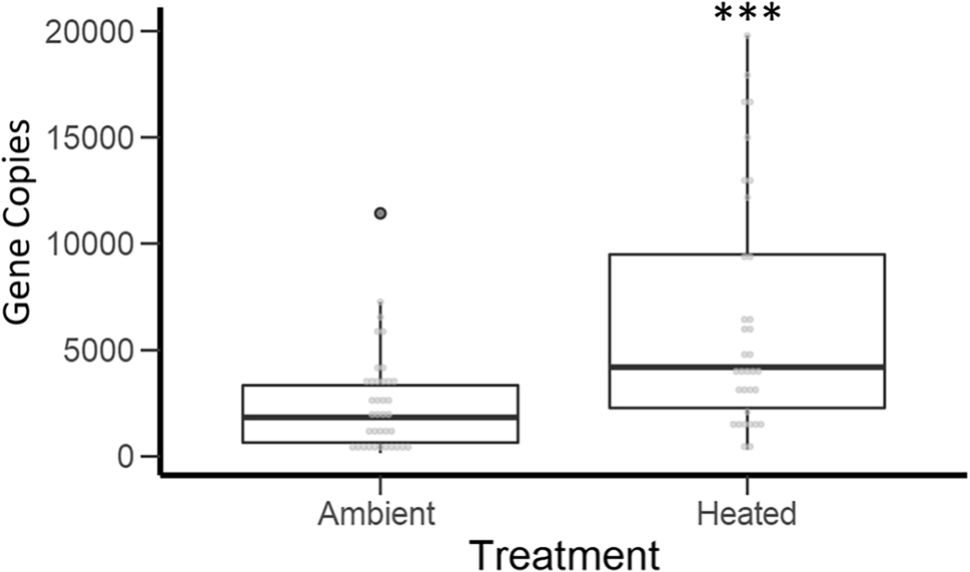
Quantitative PCR (qPCR) results showing absolute abundance of bacterial cells in each nectar sample of the different temperature treatments; there was an increase in bacterial abundance in the heated treatment. Ambient = 27 °C and heated = 32 °C. Asterisk (***) indicates statistical significance

Across all samples, a *Fructobacillus* (Leuconostocaceae) ASV was the most abundant bacterium and dominated the communities regardless of temperature treatment (Online Resource 4). As sequencing data revealed that *Fructobacillus* spp*.* dominated microbial communities in all samples, we used 16S rRNA gene qPCR to determine if the absolute abundance of bacteria differed by temperature treatment. We found significantly higher total abundance of bacteria in the 32 °C treatment compared to the 27 °C treatment (*t* =  − 3.804, df = 43.97, *p* < 0.0001; Fig. 4)*.*

## Discussion

Warming affected overall bacterial density within nectar which in turn affected nectar sugar composition and ultimately pollinator preference. Nectar sugars decreased when microbes were present, but overall sugar levels by themselves did not explain bumble bee preference. Fructose levels were lowest with bacterial inoculation at the warmest temperature, which agrees with our microbial community data. Our synthetic nectar bacterial communities were dominated by *Fructobacillus,* which were more abundant in the warmer (32 °C) incubation temperature. As its name implies, *Fructobacillus* spp. are fructophilic lactic acid bacteria that use fructose as their main carbohydrate source [41], indicating that increasing *Fructobacillus* abundance drives the decrease in fructose at the warmest temperature. *Fructobacillus* spp*.* are commonly isolated from fructose-rich environments, such as flowers and fruits, and are occasionally isolated from nectar although not as the dominant member of nectar microbial communities [41–43, 45].

Ours is the first study to characterize the microbial community of *B. rapa* nectar. While *Fructobacillus fructosus* and *F. tropaeoli* are known to inhabit flowers [44], a recent review of nectar-associated microbes did not classify *Fructobacillus* as flower specialists [45]. Most of our nectar samples also contained *Acinetobacter* and lactobacilli*,* although these bacteria accounted for fewer than 5% of sequence reads. *Acinetobacter* is frequently isolated from the nectar of many different plant species [46] and is considered a flower specialist [45]. Lactobacilli are common bee- and flower-associated bacteria that may play a role in pollinator health [47]. For example, *Apilactobacillus kunkeei* (formerly *Lactobacillus kunkeei*) has been isolated from flowers and is commonly associated with honey bee bread and may potentially aid in honey bee immunity [43, 48]. *Apilactobacillus micheneri* (formerly *Lactobacillus micheneri*) associates with flowers and non-apis bees [49, 50]. *Apilactobacillus* and *Acinetobacter* are also fructophilic bacteria and may therefore utilize fructose in our artificial nectar similarly to *Fructobacillus*. Although not detected here, the only other bacterial genus besides *Acinetobacter* that has been classified as a flower specialist is *Rosenbergiella* [45]. *Rosenbergiella nectarea* can utilize fructose and various other sugars [51] and is not considered a fructophilic bacterium. Nectar communities dominated by *Rosenbergiella* may therefore show different effects on floral chemistry and pollinator attraction under warming, warranting further study.

In contrast to the fructophilic bacteria that are often found in floral nectar, nectar-inhabiting yeasts tend to decrease overall sucrose while increasing fructose and glucose [16]. Although yeasts are commonly found in nectar their presence is not universal [52], and we did not detect them in our samples, suggesting that *B. rapa nectar* may not harbor yeasts. Dispersal of nectar microbes depends on many mechanisms including flower visitor [45], and lack of the appropriate vector or the chemistry of our artificial nectar may explain the absence of yeast in our samples. Nectar-inhabiting yeasts have the potential to warm flowers in cooler climates, which attracts pollinators [53]. However, little is known about the effects of increased environmental temperatures on floral yeasts. Future studies should examine the effects of warming on nectar microbial communities that include yeast.

The communities that we studied were dominated by a single bacterial genus, as flower microbiomes are known for low species richness and evenness [45]. Floral microbial communities are often less diverse than that of leaves and other plant parts [18]. Nectar microbial communities are filtered by nectar properties [12] making this environment hospitable mainly for a small group of flower specialists [45]. Research on priority effects indicates that the initial microbial colonist may persist and outcompete other nectar microbes later in the season [54]. However, patterns of floral microbial diversity and richness vary with geographical location [18]. The dominance of *Fructobacillus* in our lab experiment represents a “snapshot” of the *B. rapa* microbial community and may reflect a lack of diverse plant, pollinator, and microbial communities in the immediate area. As human-modified landscapes now dominate much of our planet [55], our low-diversity community may be common. Broad surveys of nectar microbial communities are still lacking [45], and continued research on this topic is needed to untangle the effects climate change has on plant-pollinator-microbe interactions.

When given the choice, bumble bees preferred nectar with bacteria at ambient temperature, suggesting that either the loss of fructose or an overabundance of microbial metabolites influenced bumble bee foraging choices. Nectar yeast metabolites, such as volatile organic compounds (VOCs), act as informative cues of nectar rewards, including indicating the presence of higher sugar concentrations [56]. Nectar with dissolved secondary metabolites from yeast elicited an enhanced gustatory response in bumble bees, indicating the importance of these chemicals [15]. Bumble bees have a strong preference towards sucrose-rich nectar [57]. As sucrose levels remained unchanged but fructose levels decreased, our results suggest that bumble bee preferences are either also affected by fructose and glucose levels or by the correct blend of sugars and microbial metabolites. It could also be that as fructose was depleted in the high-density *Fructobacillus* samples, the bacteria switched to a less preferred biochemical pathway that resulted in the formation of repulsive metabolites.

Whether sugars, bacterial metabolites, or interactions between the two drive bumble bee foraging choices needs further study. It is important to note that only the nectar bacterial communities were exposed to temperature treatments; bumble bees were kept at a constant temperature throughout the study, and we therefore do not consider changes in bee energetics with temperature. Overall, our data indicate that the microbial community within nectar is important for pollinator choice and is mediated by abiotic factors such as temperature. As temperatures increase due to climate change, alterations to nectar microbiomes may have adverse effects on pollinator choice.

As pollinators forage for resources, they use many mechanisms to choose high-quality pollen and nectar. Although high sugar concentrations are important for optimal foraging, the microbial component of nectar is also a significant factor for pollinator choice. For example, honey bees have been known to avoid nectar colonized with the bacteria *Asaia astilbes*, *Erwinia tasmaniensis*, and *Apilactobacillus kunkeei* [58]. However, honey bees are not deterred by nectar colonized with *Metschnikowia reukaufii*, a commonly found nectar-inhabiting yeast [58]. Recent studies have revealed that these yeasts have a positive impact on bumble bee colony growth [59]. Our study follows a similar pattern to this previous work, as bumble bees consumed more nectar with a bacterial community than nectar with no microbes but a higher sugar concentration. The preference for microbe-inoculated nectar indicates that either these microbes are potentially advantageous, perhaps giving nutritional benefits to the bee or that the bees are making sub-optimal foraging decisions.

We are the first to show that temperature can affect nectar-microbe-pollinator interactions in vitro. With an increase in temperature, we saw an increase in the absolute abundance of *Fructobacillus* spp*.* in the nectar. This greater density of *Fructobacillus* altered nectar chemistry and ultimately pollinator preference, connecting climate change to pollinator behavior as mediated by nectar microbes. Although we only compared two temperatures in this study there is a clear difference in bacterial abundance with temperature in vitro, and future studies should investigate the effects of a gradient of natural temperatures on this system in vivo. Previous studies have shown that climate change is affecting plant-pollinator mutualisms by causing plant phenological shifts that can disrupt pollinator mutualisms [60]. As temperatures change and precipitation decreases, plant metabolism is likely to respond to warming, altering nectar properties [61]. Alteration in nectar properties and plant response may select for different microbial communities, which in turn, may differentially affect pollinator preference. We point out that as the climate changes nectar microbes and plant physiology may continue to adapt to the new environmental conditions. Bee preference and consumption may also adapt to these potentially new nectar microbial communities and nectar chemistries, such that the results we found here may not apply to the future climate. However, bees evolve more slowly than the climate is changing [62], which suggests that disruption of these plant-pollinator-microbial interactions is likely.

## Conclusion

Our data show that warming affects the density of nectar-inhabiting microbes, which in turn alter nectar chemistry and pollinator preference. We thereby elucidate a connection between climate change, plant- and pollinator-associated microbes, and pollinator behavior. Field studies on these interactions can shed light on whether changes to nectar-inhabiting microbiomes mediated by climate change will influence pollination success and if plants are able to select nectar microbial communities under climate change stress. Future studies looking at the effects of temperature on nectar-inhabiting microbes, changes in nectar composition *in planta,* and plant fitness will be especially valuable.

## Supplementary Information

Below is the link to the electronic supplementary material.

Supplementary file1 (XLSX 51 KB)

Supplementary file2 (PDF 219 KB)

## Data Availability

Metabarcoding amplicon data and associated metadata are available on the NCBI Sequence Read Archive (SRA accession number PRJNA758089).
