# Supplementary material for: Elevated Temperature May Affect Nectar Microbes, Nectar Sugars, and Bumble Bee Foraging Preference
Source: Microb Ecol. 2021 Oct 1;84(2):473–82. doi: 10.1007/s00248-021-01881-x (PMC9436853; doi:10.1007/s00248-021-01881-x)
Supplement: Supplementary file 2 — Supplementary file2 (PDF 219 KB) [file 248_2021_1881_MOESM2_ESM.pdf]

Online Resource 1. Results from Texas A&M University Proteomics department's established plant nectar free amino acid HPLC assay.

Amino Acid	mg /ml
ASP	0.27
GLU	0.31
ASN	0.81
SER	0.46
GLN	1.68
HIS	0.60
GLY	0.28
THR	0.16
ALA	0.42
ARG	0.38
TYR	0.06
VAL	0.62
MET	0.06
TRP	0.33
PHE	0.09
ILE	0.17
LEU	0.21
LYS	0.21
PRO	1.27
Sum:	8.41

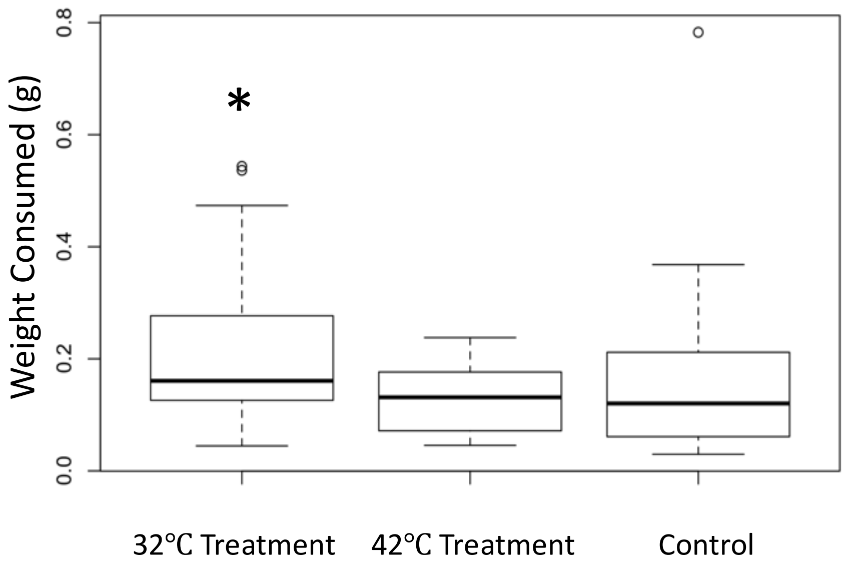

Online Resource 3. Bumble bee choice assay from preliminary experiment. Bumble bees were offered a choice between nectar inoculated with microbes an incubated at 32°C, 42°C or sterile synthetic nectar. Bumble bees consumed more nectar with a microbial community and incubated at 32°C (F= 7.1945, p = 0.00118)

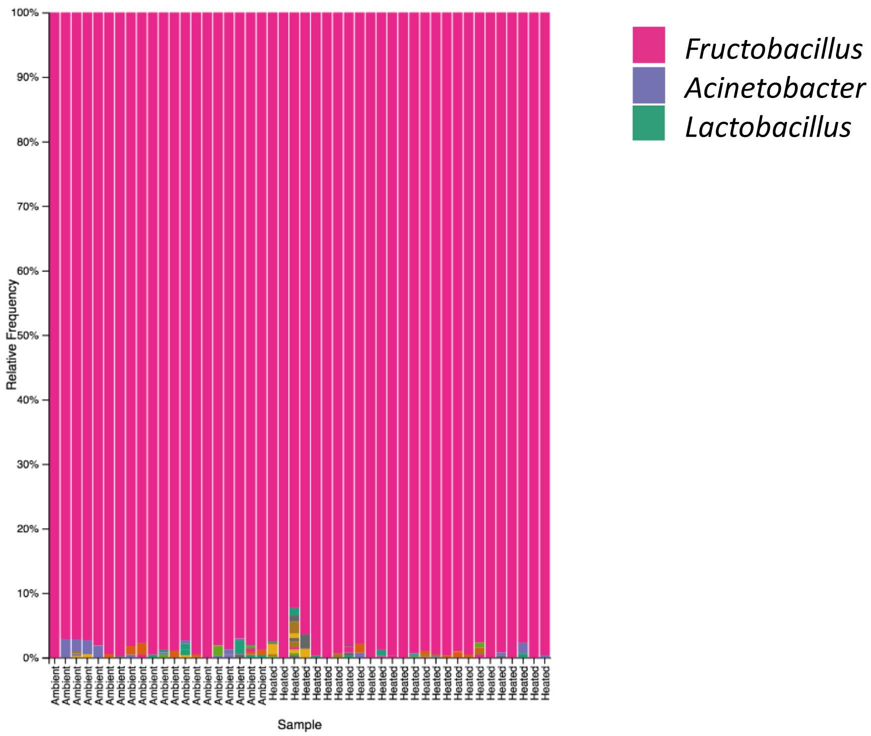

Online Resource 4. Bar graph showing relative frequency of ESVs in synthetic nectar, which were incubated at two temperature treatments: Ambient = 27°C and Heated = 32°C. Legend indicates bacterial genera found in each sample.
